# Effect of emergent nephrostomy on long-term total and split renal function in patients with upper urinary tract obstruction due to pelvic malignant tumors

**DOI:** 10.1007/s00383-024-05810-0

**Published:** 2024-08-19

**Authors:** Katsuhiro Nishimura, Ayako Takenouchi, Shugo Komatsu, Yunosuke Kawaguchi, Wataru Kudo, Shota Takiguchi, Tomoro Hishiki

**Affiliations:** Department of Pediatric Surgery, Graduate School of Medicine, Chiba University, 1-8-1 Inohana, Chiba, 260-8677 Japan

**Keywords:** Upper urinary tract obstruction, Split renal function, Nephrostomy, Childhood cancer, Pelvic malignant tumor

## Abstract

**Purpose:**

This study aimed to investigate the impact of nephrostomies on the outcome of total renal function (TRF) and split renal function (SRF) in patients with malignant pelvic tumors associated with upper urinary tract obstruction (UUTO).

**Methods:**

Patients with pelvic tumors suffering severe unilateral hydronephrosis treated at our hospital from 2000 to 2022 were included. Data for nephrostomy placement, short- and long-term renal function, and radiological and nuclear imaging studies were collected. The TRF and SRF of patients who underwent nephrostomy were compared to those who did not.

**Results:**

Seven patients were included (rhabdomyosarcoma: 5, ovarian germ cell tumor: 1, malignant rhabdoid tumor: 1). Nephrostomies were placed in four, which were successfully managed without severe infections. Estimated glomerular filtration rate (eGFR) was significantly improved at the end of treatment in patients with nephrostomy. In contrast, eGFR in patients who did not undergo nephrostomy was not improved. Nuclear imaging studies (renograms or renal scintigrams) revealed impaired SRF of the affected kidney compared to the contralateral kidney, even in patients whose eGFR was within normal levels. Notably, SRF showed a trend to improve over time in one patient treated with nephrostomy.

**Conclusion:**

Nephrostomy for UUTO caused by pelvic tumors may improve renal outcome.

**Supplementary Information:**

The online version contains supplementary material available at 10.1007/s00383-024-05810-0.

## Introduction

Upper urinary tract obstruction (UUTO) is one of the major complications associated with malignant pelvic tumors [1–3]. UUTO often causes pain and fever and reduces the quality of life of patients with malignant tumors. Bilateral UUTOs and unilateral severe UUTOs are treated as oncological emergencies due to their potential risk of acute renal failure, thus requiring emergent urinary diversions such as nephrostomy or ureteral stenting in selected cases. To date, several studies have shown that urinary diversion might effectively improve the outcome of renal function of adult patients with such conditions [4–7], but very few studies have demonstrated the impact of urinary diversion on renal function in childhood cancer [3, 8]. Furthermore, these studies have evaluated only the total renal function (TRF) of the patient using serum creatinine and estimated glomerular filtration rate (eGFR), which can be largely affected by adverse effects caused by systemic chemotherapy. The actual effect of UUTO itself, as well as urinary diversion, on the function of the affected ipsilateral kidney remains unknown.

While urinary diversion may be effective in terms of preserving renal function, nephrostomies and ureteral stents may also increase the risk of urinary tract infection (UTI), which occasionally becomes life-threatening in immunocompromised children undergoing intensive chemotherapy. Therefore, it is essential to clarify the true benefit of urinary diversion for UUTO in pediatric cancer patients to determine the optimal approach for UUTO.

This study aims to elucidate the effect of urinary diversion for UUTO associated with malignant pelvic tumors on renal function. In this study, we assessed both the total and split renal function (SRF) to clarify the impact of UUTO and urinary diversion on the affected kidney.

## Materials and methods

### Patients

In this retrospective study, patients with malignant pelvic tumors with unilateral UUTO treated at our hospital from 2000 to 2022 were included. The presence of urinary tract obstruction and hydronephrosis were assessed using computed tomography (CT) and abdominal ultrasound examination images obtained at diagnosis. We defined UUTO as the presence of grade 3 or 4 hydronephrosis according to the Society of Fetal Urology (SFU) [9]. All cases with pelvic tumors of any pathology invading or compressing the upper urinary tract were included in the study cohort. Patients with bilateral UUTO were excluded because the indications for urinary tract decompression were undoubtful for these patients. Patients showing hydronephrosis due to lower urinary tract obstruction (e.g., prostate rhabdomyosarcomas) were also excluded.

### Indication and management of nephrostomy

Percutaneous nephrostomy was favored as the primary option for urinary diversion. Decisions for placing nephrostomies were made per patient under the discretion of the multidisciplinary pediatric oncology team consisting of pediatric surgeons, pediatric oncologists, and radiologists. Percutaneous nephrostomy was performed under general anesthesia with ultrasound and fluoroscopy guidance using 8 ~ 10 Fr nephrostomy tubes according to the size of the patient. After nephrostomy placement, fluoroscopic contrast studies were performed periodically to assess whether the UUTO had been relieved in response to chemotherapy. Once the patency of the affected ureter was confirmed, the nephrostomy tube was removed after a transient occlusion test. The nephrostomy tube was changed monthly for patients with prolonged tube placement.

### Data collection

We collected the following clinical information from electronic medical records: (1) demographic data including age at diagnosis, gender, and body surface area; (2) treatment information including diagnosis, clinical stage, primary site, description of multidisciplinary treatment and outcomes; (3) hydronephrosis-specific data including SFU grade at diagnosis, interventions, complications, and outcome; (4) data on TRF, including serum creatinine levels, eGFR levels, and creatinine clearance (Ccr) levels at diagnosis and during follow-up. eGFR was calculated from a formula based on serum creatinine reference values for Japanese children by age reported by the Japanese Society for Pediatric Nephrology [10]. To evaluate the improvement in eGFR from diagnosis to the end of initial cancer treatment, we calculated the percentage change in eGFR using the following formula: (eGFR at the end of treatment − eGFR at diagnosis)/(eGFR at diagnosis) × 100 [%].

For patients who underwent nephrostomy, data on the duration of nephrostomy placement, complications related to nephrostomy, replacement with ureteral stents, and urinary diversion were extracted. In addition, SRF of the affected kidney was assessed in patients who underwent nephrostomy. For pretreatment SRF, urine obtained through the nephrostomy (representing the affected kidney) and from the bladder (representing the contralateral kidney) immediately after nephrostomy placement were used to calculate the Ccr of each kidney. To assess SRF after nephrostomies were removed, we extracted the relative radioisotope uptake values of the affected kidney from the results of nuclear imaging studies (99mTc-MAG3 diuretic renogram or 99mTc-DMSA scintigram).

### Statistical analysis

Continuous variables were summarized using the mean and standard deviation (SD) unless otherwise noted. Comparisons between the two groups were performed using Welch’s T-test. Statistical significance was defined as a two-sided P < 0.05. All statistical analyses were performed using GraphPad Prism software (version 10; GraphPad Software Inc., San Diego, CA, USA).

### Ethics

This study was performed in accordance with the ethical guidelines for medical studies in Japan and the principles of the Declaration of Helsinki. This study was approved by the Ethics Committee of Chiba University Hospital in September 2021 (M10108). The requirement for informed consent was waived because the study design was retrospective, and participant privacy was ensured.

## Results

### Patients’ characteristics

Demographic data and treatment information are shown in Table 1. Seven patients presented with unilateral UUTO caused by compression from or invasion of a malignant pelvic tumor. Three boys and four girls were identified. The median age at diagnosis was five (range: 3 to 13 years old). Histopathology of the tumors were rhabdomyosarcoma (*n* = 5), ovarian malignant germ cell tumor (*n* = 1), and malignant rhabdoid tumor (*n* = 1). All cases had advanced-stage tumors and were treated with various multimodal therapies, including surgery, chemotherapy, and radiotherapy, according to standard treatment or clinical trial protocols. Details of first-line cancer treatment are shown in Supplement Table 1. Four patients had a recurrence and were accordingly treated; two patients with rhabdomyosarcoma remained disease-free after their second remission, one with rhabdomyosarcoma was still on treatment at the time of analysis, and one with malignant rhabdoid tumor died due to progressive liver metastasis. The median follow-up period was 40 months (range: 9 to 239 months).

**Table 1:** Patients’ characteristics

Case	Sex	Year at diagnosis	Age at diagnosis (year)	Histopathology of tumor	Staging	Treatment	Recurrence	Outcome	Follow-up period (months)^1^
1	M	2001	8	RMS embryonal type	Stage 3, group III	CT, surgery, PBSCT	+	Alive without disease	239
2	M	2005	5	RMS embryonal type	Stage 3, group III	CT, surgery, PBSCT	+	Alive without disease	222
3	M	2018	4	RMS embryonal type	Stage 3, group III	CT, RT, surgery	−	Alive without disease	88
4	F	2020	13	Ovarian GCT dysgerminoma	Stage 4	CT, surgery	−	Alive without disease	40
5	F	2021	5	RMS embryonal type	Stage 3, group III	CT, RT, surgery	−	Alive without disease	33
6	F	2022	12	RMS embryonal type	Stage 3, group III	CT, RT, surgery	+	Alive with disease	28
7	F	2022	3	MRT	N.A	CT, RT, surgery	+	Died of disease	9

*M* male; *F* female; *RMS* rhabdomyosarcoma; *GCT* malignant germ cell tumor; *MRT* malignant rhabdoid tumor; *CT* chemotherapy; *PBSCT* peripheral blood stem cell transplantation; *N.A.* not available

^1^Follow-up period means time between diagnosis and the most recent visit to our hospital or death

### Nephrostomy

Details of hydronephrosis and management of nephrostomy are shown in Table 2. Six patients presented with SFU grade 3 hydronephrosis and one with SFU grade 4. Nephrostomy was placed in four patients with SFU grade 3 hydronephrosis. There was an apparent difference in the treatment policy before and after 2018; in early cases, nephrostomy was avoided considering the potential risk of uncontrollable UTI, and contrarily, all cases treated after 2018 were subjected to immediate nephrostomy. Percutaneous nephrostomy was successfully performed in all patients. Complications after nephrostomy placement included a displaced nephrostomy tube in one case, which required reinsertion 1 week after the initial surgery. No patients developed febrile UTIs during nephrostomy management. The placement of nephrostomy did not cause any significant delay in chemotherapy. The median duration of nephrostomy placement was 87 days (range 18 to 256 days). In the two cases (cases 4 and 5) in which contrast studies confirmed ureteral patency, the nephrostomy tube was removed before tumor resection. In the other two cases (cases 6 and 7), the nephrostomy tube remained in place until or beyond tumor resection because of residual ureteral stenosis or obstruction due to the tumor. In case 6, which demonstrated complete obstruction of the ureter, the ureter was partially resected en bloc with the tumor due to invasion. The ureter was reconstructed via a transureteroureterostomy to the contralateral ureter. In all four patients, a double-J catheter was placed during tumor resection to prevent ureteral injury or as a stent through the anastomosis. Only one case (case 6) developed a febrile UTI while the double-J catheter was in place. The catheter was removed early postoperatively in this patient.

**Table 2:** Details of hydronephrosis and management of nephrostomy

Case	SFU grade at diagnosis	Nephrostomy	Nephrostomy-related complications	Duration of nephrostomy placement (days)
1	4	−	N.A	N.A
2	3	−	N.A	N.A
3	3	−	N.A	N.A
4	3	+	–	18
5	3	+	–	31
6	3	+	Displaced nephrostomy tube	256
7	3	+	–	143

*SFU* the Society of Fetal Urology; *N.A.* not applicable

### TRF assessed by eGFR

**Fig. 1:**
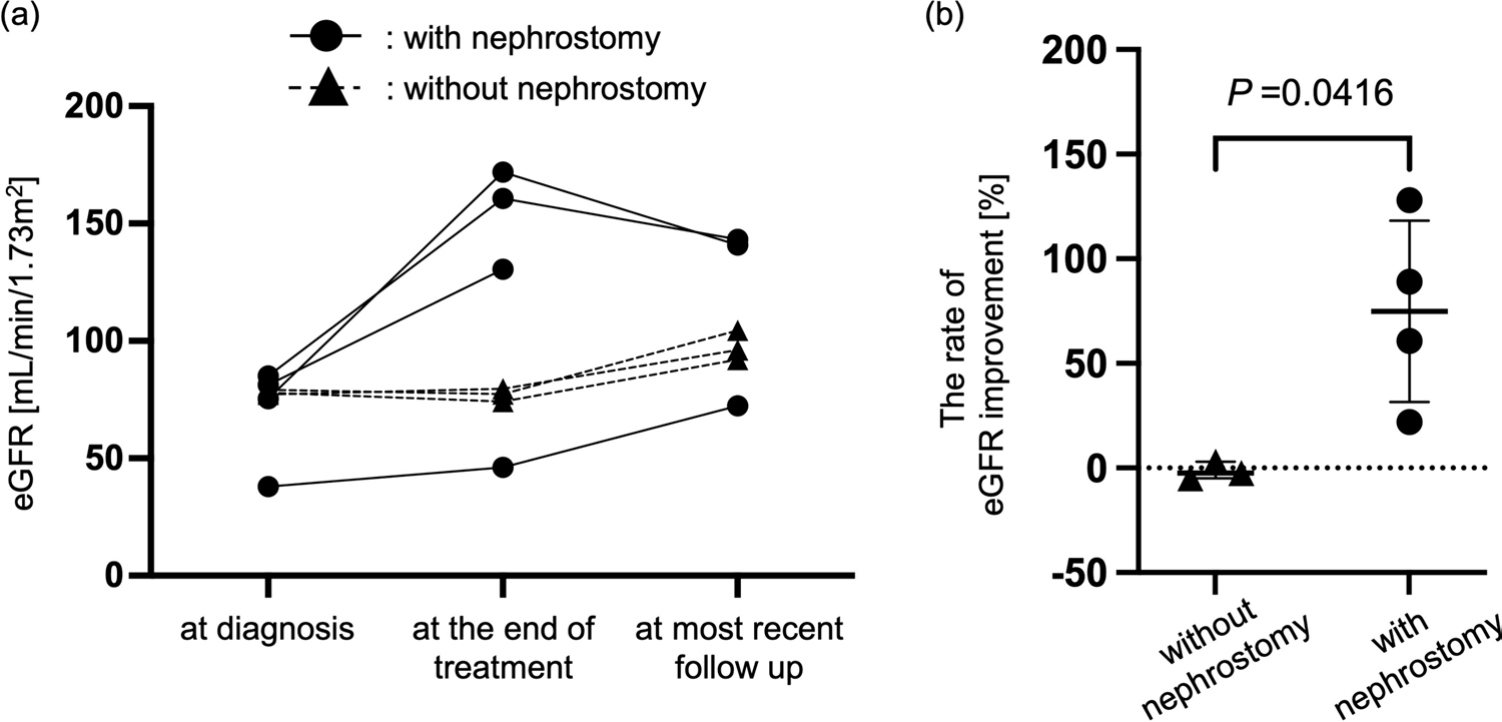
Total renal function assessed by eGFR. **a** Changes in eGFR from diagnosis to the most recent follow-up. Dot plots represent eGFR value for each patient. **b** Percentage change in eGFR from diagnosis to the end of initial cancer treatment. The rate of eGFR improvement is calculated using the formula: (eGFR at the end of treatment − eGFR at diagnosis)/(eGFR at diagnosis) × 100 [%]. Error bars indicate the mean with standard deviation. *P*, *P* value

The outcome of TRF assessed by eGFR is shown in Fig. 1. Three of the four patients who underwent nephrostomy had improved eGFR at the end of multidisciplinary treatment compared to the baseline value at diagnosis (69.9 ± 21.9 versus 127.4 ± 56.8 mL/min/1.73 m^2^). In contrast, the three patients who did not undergo nephrostomy showed no significant change in eGFR values from diagnosis to the end of multidisciplinary treatment (78.1 ± 1.0 versus 77.0 ± 2.7 mL/min/1.73 m^2^). The rate of eGFR improvement was significantly greater in patients undergoing nephrostomy than those who did not (− 1.39 ± 4.09 versus 74.9 ± 44.8%, P = 0.0416). In one of the three patients without nephrostomies, the affected kidney was surgically removed concurrently with the tumor. In two other patients, the affected kidneys remained in a UUTO status and eventually became severely atrophic.

### SRF in patients who underwent nephrostomy

Among the four patients who underwent nephrostomy, data for 24-h Ccr of each kidney immediately after nephrostomy placement was available in three cases. In these cases, urine produced by the affected kidney was collected through the nephrostomy, while urine from the contralateral unaffected kidney was collected from the bladder, and both were subjected to Ccr calculation (Table 3). The Ccr of the affected kidney was lower than that of the contralateral kidney in all three cases, although not statistically significant (affected versus contralateral: 14.4 ± 12.5 versus 132.2 ± 65.2 mL/min) (P = 0.0609).

**Table 3:** Ccr of affected and contralateral kidney at diagnosis

Case	24-h Ccr [mL/min]	
	Affected kidney	Contralateral kidney
4	3.06	67.1
5	N.A	N.A
6	12.2	132.2
7	27.8	197.6

*Ccr* creatinine clearance; *N.A.* not available

Data from 99mTc-MAG3 diuretic renogram or 99mTc-DMSA renal scintigram studies during or after treatment were available in three of the four patients initially treated with nephrostomy, and SRF was assessed. We found that relative radioisotope uptake of the affected kidney as measured by 99mTc-MAG3 diuretic renogram was at least 10% lower than the contralateral unaffected kidney after completion of first-line cancer treatment in all three cases (affected versus contralateral; case 4: 8.5% versus 91.5%, case 5: 33% versus 67%, case 6: 40% versus 60%). This trend was evident even in cases 5 and 6, whose eGFR was in the normal range after completion of first-line cancer treatment (case 5: 171.9 mL/min/1.73 m^2^, case 6: 160.7 mL/min/1.73 m^2^). We were able to evaluate the longitudinal changes of the SRF over time of case 4, which demonstrated mild atrophy of the affected kidney. The SRF at the completion of treatment and at 1 year after completion was assessed using 99mTc-MAG3 diuretic renogram, whereas 99mTc-DMSA renal scintigram was used to assess SRF at 2 years after completion of treatment. This patient showed a relatively low relative radioisotope uptake at the end of treatment (8.5%) but showed a trend toward improvement over time (17.7% at one year and 23.6% at 2 years after completion of treatment).

## Discussion

This retrospective study investigated the effect of nephrostomy for UUTO due to malignant pelvic tumors on the prognosis of total and split renal function. The principal findings of our study were as follows. First, patients with nephrostomy had improved eGFR at the end of multidisciplinary treatment compared to the time of diagnosis; this trend was not observed in patients without nephrostomies. Second, patients with apparently normal eGFR levels had potentially impaired split function of the affected kidney. Third, impaired SRF may be improved over time after the completion of treatment.

Several previous studies have assessed the relationship between urinary diversion and TRF for UUTO associated with malignant solid tumors [3, 4, 6]. Alexander et al. reported that 19 of 53 pediatric patients with malignant solid tumors presenting with hydronephrosis had residual UUTO after primary treatment such as chemotherapy and surgery. Seven of these 19 patients underwent urinary diversion, and 6 had improved serum creatinine levels [3]. Aravantinos et al. described that 270 adult patients with advanced-stage cancer who presented with obstructive nephropathy all had an average improvement in serum creatinine levels of approximately 4 mg/dL after nephrostomy [4]. These previous studies supported our findings which showed improved eGFR levels at the end of treatment compared to the baseline assessment at diagnosis in patients treated with nephrostomy.

In addition, the strength of our findings is that we evaluated SRF in addition to TRF in patients undergoing nephrostomy to assess the direct effect of UUTO and nephrostomy on the affected kidney. Our results indicated that the SRF of the affected kidney had already been severely impaired at diagnosis due to the presence of UUTO and remained impaired until the completion of first-line cancer treatment. Impaired SRF was evident even in cases whose eGFR appeared to be normal. Thus, renal function analysis using eGFR may be insufficient to capture the potentially impaired function of the affected kidney. Although urinary division is accepted as a promising procedure to rescue the renal function of patients with UUTO associated with malignancy, no publications to date have demonstrated the impact of nephrostomy on the SRF. Our results suggest the positive effect of emergent urinary diversion on the affected kidney. Considering the rapid growth of pediatric solid tumors in general, it is likely that these obstructions had been formed in a relatively short period, and urgent relief of the obstruction may at least partially recover the function of the obstructed kidney.

While nephrostomy and ureteral stenting for malignant UUTO may improve renal function, they also carry potential risks for causing UTI. The risk of severe infections is particularly high in children receiving intensive chemotherapy, which can lead to uncontrollable sepsis as the most extreme consequence. It can also significantly delay the time course of systemic chemotherapy, which could also affect the oncological outcome. Bahu et al. reported that 38 of 200 patients with percutaneous nephrostomy for UUTO due to malignancy developed nephrostomy-related UTI, and a history of UTI and neutropenia were found to be significant risk factors for pyelonephritis[11]. Although the number of cases in our series was small, we did not experience nephrostomy-related UTI in the studied cohort. The low frequency of UTIs may be due to the fact that the tubes were periodically replaced and were managed under meticulous care. Several studies have shown that periodic replacement of nephrostomy tubes and appropriate antimicrobials reduce the risk of UTI [12–15]. Periodic replacement at 60 to 90 days is suggested to prevent UTI and nephrostomy obstruction in nonmalignant conditions [12, 13]. Usage of prophylactic antimicrobials is controversial and is not uniformly recommended [11, 15, 16]. Szvalb et al. suggested immediate catheter replacement within 4 days of infection and continuation of appropriate antimicrobial therapy for 10–14 days to prevent the recurrence of nephrostomy-related UTI [14]. Alma et al. reported that the most important predisposing factors to prevent UTI were the patient and/or the caregiver of the patient being trained in catheter care [15].

The choice of nephrostomies or ureteral stenting as a tool for urinary division remains controversial. The failure rate of retrograde ureteral stents for UUTOs caused by non-urinary malignancies is reportedly 15–40% [17–19]. Obstruction of the middle and lower ureter is considered a predictor of stent failure [20]. In case 6, we attempted to insert a retrograde ureteral stent, but resulted in failure due to severe stenosis of the lower ureter. The other cases were not candidates for endoscopic stenting because of severe deformity of the bladder due to compression from the tumor. In a study evaluating the effect of nephrostomy or double-J stenting for UUTO caused by pediatric pelvic tumors, Meir et al. reported nephrostomy-related infections in 3 of 12 patients whose nephrostomies were implanted for over 6 weeks. They recommend placing a nephrostomy for initial diversion but converting to a double-J stent placement if long-term retention is anticipated [8]. Contrarily, none of our patients had a UTI during nephrostomy tube placement, but one patient suffered a UTI after replacing the initial nephrostomy with a double-J stent.

The current study has several limitations. First, the study is a retrospective analysis using a relatively small sample, limiting the inferencing of any causality. As a nature of a retrospective study, SRF was evaluated using various methods at random timings and lacked consistency. All evaluations were done for treatment purposes, and the timing of the assessments was determined at the treating teams’ discretion. Second, the effects of chemotherapy and radiotherapy on TRF have not been taken into consideration. Differences in eGFR value between nephrostomy and non-nephrostomy patients may be significantly affected by the differences in chemotherapy regimens, period of treatment, or radiation doses. These factors could be a significant bias in the assessment of renal function. Third, the follow-up period for nephrostomy cases was relatively short, and the long-term outcome of renal function remains unclear. Finally, nuclear imaging studies were not obtained before nephrostomy placement in any of our patients due to the urgent conditions of the patients, so we could not directly compare the SRF before and after the placement of nephrostomies. Notably, in case 4, Ccr of the affected kidney accounted for only 4.3% of the total Ccr at the initial evaluation immediately after nephrostomy placement, while the SRF measured by renograms after the completion of the cancer treatment was improved to 8.5%. A similar finding was observed in case 6, where the initial split Ccr accounted for 8.4% but was recovered to 40% in a post-treatment renogram. The SRF after the completion of treatment in case 4 had been measured with different nuclear imaging studies. Since previous studies have shown that SRF measured by 99mTc-MAG3 and 99mTc-DMSA renal scintigram are comparable [21, 22], we consider that our results are sufficient to suggest that the SRF of case 4 improved over time after the completion of treatment. Even though we could not directly compare the SRF measured with different modalities, we may speculate that nephrostomy directly affected the function of the affected kidney through these findings.

A validation study, preferably conducted in a prospective manner and assessing immediate and long-term outcomes of UUTOs treated with nephrostomies using a larger cohort, is warranted.

## Conclusion

Nephrostomy for UUTO may contribute to improving TRF and SRF in children with malignant pelvic solid tumors.

## Supplementary Information

Below is the link to the electronic supplementary material.

Supplementary file1 (DOCX 30.3 kb)

## Data Availability

No datasets were generated or analysed during the current study.
