# Supplementary material for: Effect of emergent nephrostomy on long-term total and split renal function in patients with upper urinary tract obstruction due to pelvic malignant tumors
Source: Pediatr Surg Int. 2024 Aug 19;40(1):234. doi: 10.1007/s00383-024-05810-0 (PMC11333509; doi:10.1007/s00383-024-05810-0)
Supplement: Supplementary file 1 — Supplementary file1 (DOCX 30.3 kb) [file 383_2024_5810_MOESM1_ESM.docx]

Supplement Table 1: Details of first-line cancer treatment

|  | chemotherapy | radiotherapy | | | | | total duration of first-line treatment (months) |
| --- | --- | --- | --- | --- | --- | --- | --- |
| Case | regimen | radiation type | irradiation area | irradiation to kidney | irradiation  to bladder | total radiation dose |  |
| 1 | VAC-THP, IVA, CDDP+ACT-D, Hi-MEC | N.A. | N.A. | N.A. | N.A. | N.A. | 13 |
| 2 | VAC-THP, IVA, CDDP+ACT-D, Hi-MEC | N.A. | N.A. | N.A. | N.A. | N.A. | 12 |
| 3 | VAC, VI, TC | PB | tumor bed | - | - | 50.4 Gy | 16 |
| 4 | BEP | N.A. | N.A. | N.A. | N.A. | N.A. | 4 |
| 5 | VAC, VI | PB | tumor bed | - | - | 41.4 Gy | 9 |
| 6 | VAC, VI | PB | tumor bed | - | - | 50.4 Gy | 12 |
| 7 | VDC, VI, IE | XR (IMRT) | liver metastases | - | - | 19.8 Gy | 8 |

VAC, vincristine, actinomycin D, cyclophosphamide; THP, THP-adriamycin; IVA, ifosfamide, vincristine, actinomycin D; CDDP, cisplatin; ACT-D, actinomycin D; Hi-MEC, melphalan, etoposide, carboplatin; VI, vincristine, irinotecan; TC, paclitaxel, carboplatin; BEP, bleomycin, etoposide, cisplatin; VDC/IE, vincristine, doxorubisin, cyclophosphamide, irinotecan, etoposide; PB, proton beam; XR, x-ray; IMRT, Intensity Modulated Radiation Therapy; N.A., not applicable
